# Supplementary material for: How to Deal with Second Line Dilemma in Metastatic Colorectal Cancer? A Systematic Review and Meta-Analysis
Source: Cancers (Basel). 2019 Aug 15;11(8):1189. doi: 10.3390/cancers11081189 (PMC6721481; doi:10.3390/cancers11081189)
Supplement: Supplementary file 1 [file cancers-11-01189-s001.pdf]

TableS1. Characteristics of trials included in our indirect comparisons.

Study (reference)	n1/n2	Drug	ORR n.(%)	DCR n(%)	PFS HR (95% CI)	OS HR (95% CI)
Giantonio et al 2007	286/291	Folfox + Bevacizumab<br>Folfox	65/286(22.7%)<br>25/291(8.6%)	N/A	0.61 (0.48-0.78)	0.75 (0.63-0.89)
Bennouna et al 2013	409/411	Doublet + Bevacizumab<br>Doublet	22/404 (6%)<br>16/406 (4%)	275/404 (69%)<br>220/406 (54%)	0.68 (0.59-0.58)	0.81 (0.69-0.94)
Masi et al 2015	92/92	Doublet + Bevacizumab<br>Doublet	19/92 (21%)<br>16/92 (17%)	64/92 (70%)<br>53/92 (58%)	0.70 (0.52-0.95)	0.77 (0.56-1.06)
Van Cutsem et al 2012	612/614	Folfiri + Aflibercept<br>Folfiri	105/531 (19.8%)<br>59/530(11.2%)	455/531(85.7%)<br>403/530(76%)	0.76 (0.67-0.87)	0.82 (0.71-0.94)
Tabernero et al 2015	536/536	Folfiri + Ramucirumab<br>Folfiri	72/536 (13.4%)<br>67/536 (12.5%)	397/536(74.1%)<br>369/536(68.8%)	0.79 (0.70-0.90)	0.84 (0.73-0.98)
Sobrero et al 2008	648/650	Irinotecan+Cetuximab<br>Irinotecan	106/648(16.4%)<br>27/650 (4.2%)	398/648 (61.5%)<br>298/650 (45.9%)	0.69 (0.62-0.78)	0.97 (0.85-1.11)
Peeters et al 2010	591/595	Folfiri+Panitumumab<br>Folfiri	134/529(25.3%)<br>61/522(11.7%)	378/529(71.5%)<br>334/522(64%)	N/A	N/A
Seymour et al 2013	230/230	Irinotecan+Panitumumab<br>Paniumumab	79/230(34.3%)<br>27/230(11.7%)	135/230(58.7%)<br>118/230(51.3%)	0.78(0.64-0.95)	1.01(0.83-1.23)

Study (reference)	KRAS WT/mut	PFS (RAS WT) HR(95% CI)	PFS (RAS Mut) HR(95% CI)	OS (RAS WT) HR(95% CI)	OS (RAS Mut) HR(95% CI)	ORR (RAS WT) n.(%)	ORR (RAS Mut) n.(%)
Giantonio et al 2007	N/A	N/A	N/A	N/A	N/A	N/A	N/A
Bennouna et al 2013	151/164<br>165/136	0.61 (0.49-0.77)	0.70 (0.56-0.89)	0.69 (0.53-0.90)	0.92 (0.71-1.18)	11/151 (7.3%)<br>9/164(5.5%)	6/164(3.7%)<br>4/134 (3%)
Masi et al 2015	32/40<br>36/32	N/A	N/A	N/A	N/A	N/A	N/A
Van Cutsem et al 2012	218/264	0.67(0.49-0.93)	0.80(0.60-1.07)	0.70(0.50-0.97)	0.93(0.7-1.23)	N/A	34/264(12.9%)<br>36/264(13.6%)
Tabernero et al 2015	267/269<br>275/261	0.77(0.65-0.92)	084(0.70-1.00)	0.82(0.67-1.00)	0.89(0.73-1.09)	N/A	N/A
Sobrero et al 2008	N/A	N/A	N/A	N/A	N/A	N/A	N/A
Peeters et al 2010	303/238<br>294/248	0.70 (0.54-0.91)	0.86 (0.71-1.05)	0.81 (0.63-1.03)	0.91 (0.76-1.10)	105/297 (35%)<br>28/285 (10%)	30/232 (13%)<br>33/237 (14%)
Seymour et al 2013	N/A	0.68(0.53-0.87)	1.20(0.83-1.74)	0.92(0.73-1.16)	1.64(1.15-2.35)	70/160(43.8%)<br>20/163(12.3%)	9/70(12.9%)<br>7/67(10.4%)

**Study (reference)**	**DCR (RAS WT) n.(%)**	**DCR (RAS Mut) n.(%)**	**BRAF WT/mut**	**OS BRAF WT HR(95% CI)**	**OS BRAF Mut HR(95% CI)**	**PFS BRAF WT HR(95% CI)**	**PFS BRAF Mut HR(95% CI)**
Giantonio et al 2007	N/A	N/A	N/A	N/A	N/A	N/A	N/A
Bennounna et al 2013	110/151 (72,85%) 100/164 (61%)	114/164 (69,5%) 75/134 (56%)	N/A	N/A	N/A	N/A	N/A
Masi et al 2015	N/A	N/A	65/7 64/3	N/A	N/A	N/A	N/A
Van Cutsem et al 2012	N/A	N/A	446/36	0.84(0.67-1.05)	0.42(0.16-1.09)	0.76(0.61-0.94)	0.59(0.22-1.58)
Tabernero et al 2015	N/A	N/A	N/A	N/A	N/A	N/A	N/A
Sobrero et al 2008	N/A	N/A	N/A	N/A	N/A	N/A	N/A
Peeters et al 2010	220/297 (74%) 185/285 (65%)	162/232 (68%) 149/237 (63%)	186/22 190/23	0.83(0.70-0.98)	0.64(0.32-1.28)	0.68(0.51-0.90)	0.69(0.32-1.49)
Seymour et al 2013	N/A	N/A	N/A	N/A	1.84(1.16-2.92)	N/A	1.40(0.82-2.39)

FigureS2

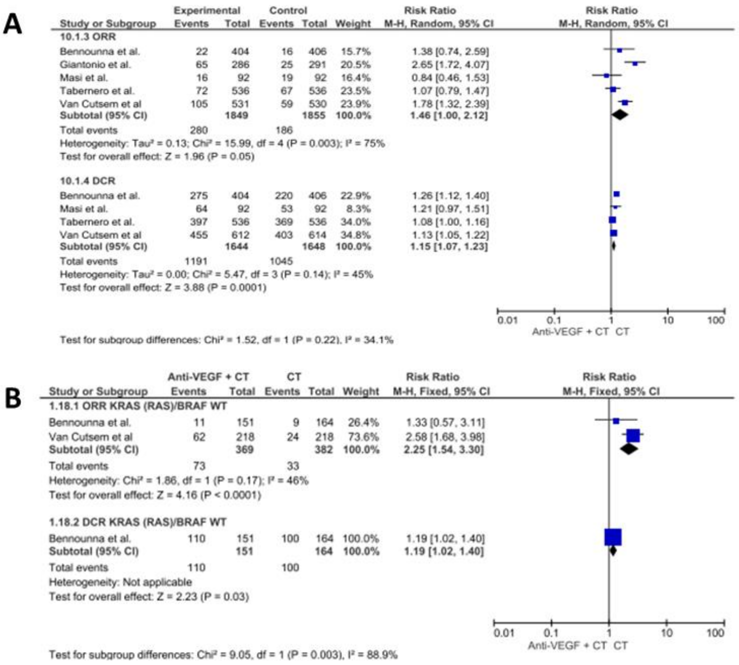
A
Experimental
Control
Risk Ratio
Risk Ratio
Study or Subgroup
Events
Total
Events
Total
Weight
M-H, Random, 95% CI
M-H, Random, 95% CI
10.1.3 ORR
Bennounna et al. 22 404 16 406 15.7% 1.38 [0.74, 2.59]
Giantonio et al. 65 286 25 291 20.5% 2.65 [1.72, 4.07]
Masi et al. 16 92 19 92 16.4% 0.84 [0.46, 1.53]
Tabernero et al. 72 536 67 536 23.5% 1.07 [0.79, 1.47]
Van Cutsem et al 105 531 59 530 23.9% 1.78 [1.32, 2.39]
Subtotal (95% CI) 1849 1855 100.0% 1.46 [1.00, 2.12]
Total events 280 186
Heterogeneity: Tau² = 0.13; Chi² = 15.99, df = 4 (P = 0.003); I² = 75%
Test for overall effect: Z = 1.96 (P = 0.05)
10.1.4 DCR
Bennounna et al. 275 404 220 406 22.9% 1.26 [1.12, 1.40]
Masi et al. 64 92 53 92 8.3% 1.21 [0.97, 1.51]
Tabernero et al. 397 536 369 536 34.0% 1.08 [1.00, 1.16]
Van Cutsem et al 455 612 403 614 34.8% 1.13 [1.05, 1.22]
Subtotal (95% CI) 1644 1648 100.0% 1.15 [1.07, 1.23]
Total events 1191 1045
Heterogeneity: Tau² = 0.00; Chi² = 5.47, df = 3 (P = 0.14); I² = 45%
Test for overall effect: Z = 3.88 (P = 0.0001)
0.01
0.1
1
10
100
Anti-VEGF + CT CT
Test for subgroup differences: Chi² = 1.52, df = 1 (P = 0.22), I² = 34.1%
B
Anti-VEGF + CT
CT
Risk Ratio
Risk Ratio
Study or Subgroup
Events
Total
Events
Total
Weight
M-H, Fixed, 95% CI
M-H, Fixed, 95% CI
1.18.1 ORR KRAS (RAS)/BRAF WT
Bennounna et al. 11 151 9 164 26.4% 1.33 [0.57, 3.11]
Van Cutsem et al 62 218 24 218 73.6% 2.58 [1.68, 3.98]
Subtotal (95% CI) 369 382 100.0% 2.25 [1.54, 3.30]
Total events 73 33
Heterogeneity: Chi² = 1.86, df = 1 (P = 0.17); I² = 46%
Test for overall effect: Z = 4.16 (P < 0.0001)
1.18.2 DCR KRAS (RAS)/BRAF WT
Bennounna et al. 110 151 100 164 100.0% 1.19 [1.02, 1.40]
Subtotal (95% CI) 151 164 100.0% 1.19 [1.02, 1.40]
Total events 110 100
Heterogeneity: Not applicable
Test for overall effect: Z = 2.23 (P = 0.03)
0.01
0.1
1
10
100
Anti-VEGF + CT CT
Test for subgroup differences: Chi² = 9.05, df = 1 (P = 0.003), I² = 88.9%

FigureS3

A

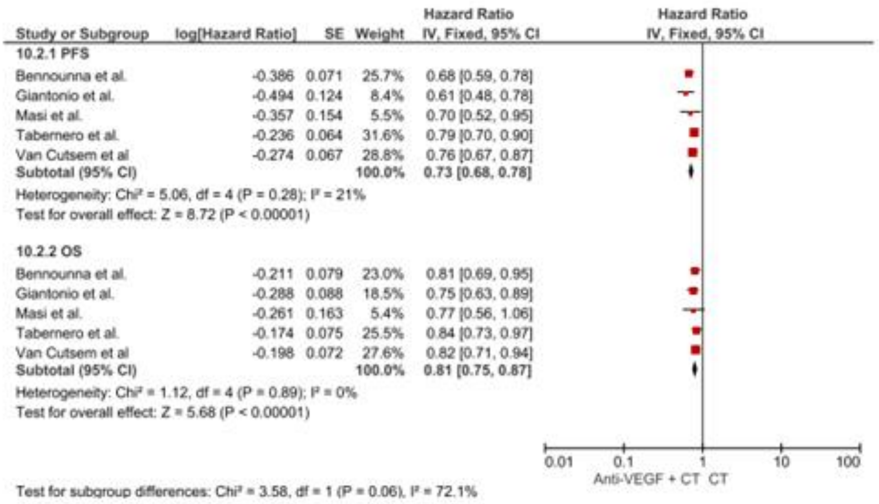

Hazard Ratio
Hazard Ratio
Study or Subgroup log[Hazard Ratio] SE Weight IV, Fixed, 95% CI
IV, Fixed, 95% CI
10.2.1 PFS
Bennounna et al. -0.386 0.071 25.7% 0.68 [0.59, 0.78]
Giantonio et al. -0.494 0.124 8.4% 0.61 [0.48, 0.78]
Masi et al. -0.357 0.154 5.5% 0.70 [0.52, 0.95]
Tabernero et al. -0.236 0.064 31.6% 0.79 [0.70, 0.90]
Van Cutsem et al -0.274 0.067 28.8% 0.76 [0.67, 0.87]
Subtotal (95% CI) 100.0% 0.73 [0.68, 0.78]
Heterogeneity: Chi² = 5.06, df = 4 (P = 0.28); I² = 21%
Test for overall effect: Z = 8.72 (P < 0.00001)
10.2.2 OS
Bennounna et al. -0.211 0.079 23.0% 0.81 [0.69, 0.95]
Giantonio et al. -0.288 0.088 18.5% 0.75 [0.63, 0.89]
Masi et al. -0.261 0.163 5.4% 0.77 [0.56, 1.06]
Tabernero et al. -0.174 0.075 25.5% 0.84 [0.73, 0.97]
Van Cutsem et al -0.198 0.072 27.6% 0.82 [0.71, 0.94]
Subtotal (95% CI) 100.0% 0.81 [0.75, 0.87]
Heterogeneity: Chi² = 1.12, df = 4 (P = 0.89); I² = 0%
Test for overall effect: Z = 5.68 (P < 0.00001)
0.01 0.1 1 10 100
Anti-VEGF + CT CT
Test for subgroup differences: Chi² = 3.58, df = 1 (P = 0.06), I² = 72.1%

B

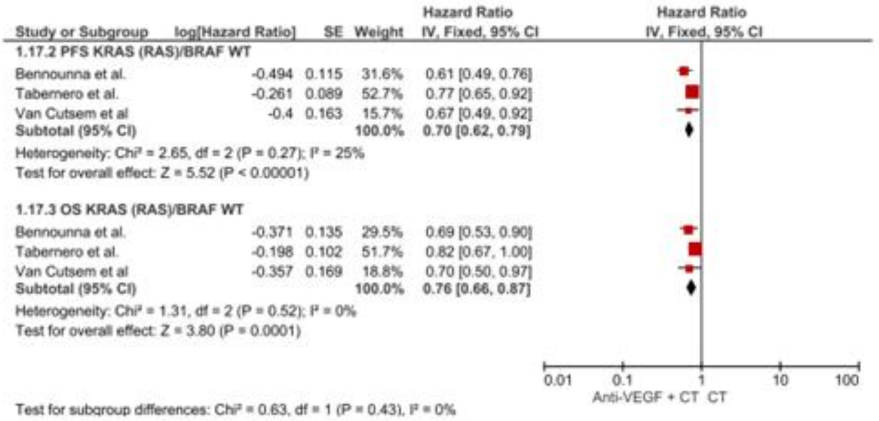

Hazard Ratio
Hazard Ratio
Study or Subgroup log[Hazard Ratio] SE Weight IV, Fixed, 95% CI
IV, Fixed, 95% CI
1.17.2 PFS KRAS (RAS)/BRAF WT
Bennounna et al. -0.494 0.115 31.6% 0.61 [0.49, 0.76]
Tabernero et al. -0.261 0.089 52.7% 0.77 [0.65, 0.92]
Van Cutsem et al -0.4 0.163 15.7% 0.67 [0.49, 0.92]
Subtotal (95% CI) 100.0% 0.70 [0.62, 0.79]
Heterogeneity: Chi² = 2.65, df = 2 (P = 0.27); I² = 25%
Test for overall effect: Z = 5.52 (P < 0.00001)
1.17.3 OS KRAS (RAS)/BRAF WT
Bennounna et al. -0.371 0.135 29.5% 0.69 [0.53, 0.90]
Tabernero et al. -0.198 0.102 51.7% 0.82 [0.67, 1.00]
Van Cutsem et al -0.357 0.169 18.8% 0.70 [0.50, 0.97]
Subtotal (95% CI) 100.0% 0.76 [0.66, 0.87]
Heterogeneity: Chi² = 1.31, df = 2 (P = 0.52); I² = 0%
Test for overall effect: Z = 3.80 (P = 0.0001)
0.01 0.1 1 10 100
Anti-VEGF + CT CT
Test for subgroup differences: Chi² = 0.63, df = 1 (P = 0.43), I² = 0%

FigureS4

Study or Subgroup	Anti-VEGF + CT Events	Anti-VEGF + CT Total	CT Events	CT Total	Weight	Risk Ratio M-H, Random, 95% CI
**1.15.1 SAE Grade 3-5 total**						
Bennounna et al.	255	401	235	409	8.5%	1.11 [0.99, 1.24]
Giantonio et al.	215	287	174	285	8.5%	1.23 [1.09, 1.38]
Masi et al.	39	91	40	92	6.2%	0.99 [0.71, 1.37]
Tabernero et al.	418	529	329	528	8.7%	1.27 [1.17, 1.37]
Van Cutsem et al	510	611	378	605	8.8%	1.34 [1.24, 1.43]
**Subtotal (95% CI)**		**1919**		**1919**	**40.7%**	**1.23 [1.14, 1.33]**
Total events	1437		1156			
Heterogeneity: Tau² = 0.00; Chi² = 10.31, df = 4 (P = 0.04); I² = 61%						
Test for overall effect: Z = 5.15 (P < 0.00001)						
**1.15.2 Bleeding**						
Bennounna et al.	8	401	1	409	0.5%	8.16 [1.03, 64.94]
Giantonio et al.	10	287	1	285	0.5%	9.93 [1.28, 77.07]
Masi et al.	0	91	0	92		Not estimable
Tabernero et al.	13	529	9	528	2.3%	1.44 [0.62, 3.34]
Van Cutsem et al	18	611	10	605	2.7%	1.78 [0.83, 3.83]
**Subtotal (95% CI)**		**1919**		**1919**	**6.0%**	**2.40 [1.11, 5.23]**
Total events	49		21			
Heterogeneity: Tau² = 0.24; Chi² = 5.04, df = 3 (P = 0.17); I² = 40%						
Test for overall effect: Z = 2.21 (P = 0.03)						
**1.15.3 Hypertension**						
Bennounna et al.	7	401	5	409	1.4%	1.43 [0.46, 4.46]
Giantonio et al.	18	287	5	285	1.8%	3.57 [1.35, 9.50]
Masi et al.	2	91	1	92	0.4%	2.02 [0.19, 21.91]
Tabernero et al.	59	529	15	528	4.0%	3.93 [2.26, 6.83]
Van Cutsem et al	118	611	9	605	3.2%	12.98 [6.65, 25.33]
**Subtotal (95% CI)**		**1919**		**1919**	**10.9%**	**4.07 [1.82, 9.09]**
Total events	204		35			
Heterogeneity: Tau² = 0.55; Chi² = 15.01, df = 4 (P = 0.005); I² = 73%						
Test for overall effect: Z = 3.42 (P = 0.0006)						
**1.15.4 Neutropenia**						
Bennounna et al.	65	401	52	409	6.1%	1.27 [0.91, 1.79]
Masi et al.	29	91	28	92	5.1%	1.05 [0.68, 1.61]
Tabernero et al.	203	529	123	528	7.8%	1.65 [1.36, 1.99]
Van Cutsem et al	224	611	178	605	8.1%	1.25 [1.06, 1.46]
**Subtotal (95% CI)**		**1632**		**1634**	**27.2%**	**1.34 [1.11, 1.61]**
Total events	521		381			
Heterogeneity: Tau² = 0.02; Chi² = 6.65, df = 3 (P = 0.08); I² = 55%						
Test for overall effect: Z = 3.07 (P = 0.002)						
**1.15.5 Venous thromboembolism**						
Bennounna et al.	19	401	12	409	3.0%	1.61 [0.79, 3.28]
Masi et al.	1	91	3	92	0.4%	0.34 [0.04, 3.18]
Tabernero et al.	22	529	11	528	2.9%	2.00 [0.98, 4.08]
Van Cutsem et al	48	611	38	605	5.3%	1.25 [0.83, 1.89]
**Subtotal (95% CI)**		**1632**		**1634**	**11.6%**	**1.40 [1.02, 1.92]**
Total events	90		64			
Heterogeneity: Tau² = 0.00; Chi² = 2.94, df = 3 (P = 0.40); I² = 0%						
Test for overall effect: Z = 2.11 (P = 0.03)						
**1.15.6 Proteinuria**						
Giantonio et al.	2	287	0	285	0.2%	4.97 [0.24, 102.97]
Masi et al.	28	91	0	92	0.3%	57.62 [3.57, 929.81]
Tabernero et al.	16	529	1	528	0.5%	15.97 [2.13, 119.99]
Van Cutsem et al	48	611	7	605	2.6%	6.79 [3.10, 14.89]
**Subtotal (95% CI)**		**1518**		**1510**	**3.6%**	**8.48 [4.20, 17.13]**
Total events	94		8			
Heterogeneity: Tau² = 0.01; Chi² = 3.03, df = 3 (P = 0.39); I² = 1%						
Test for overall effect: Z = 5.96 (P < 0.00001)						
**Total (95% CI)**		**10539**		**10535**	**100.0%**	**1.62 [1.39, 1.88]**
Total events	2395		1665			
Heterogeneity: Tau² = 0.06; Chi² = 149.16, df = 25 (P < 0.00001); I² = 83%						
Test for overall effect: Z = 6.30 (P < 0.00001)						
Test for subgroup differences: Chi² = 39.84, df = 5 (P < 0.00001), I² = 87.4%						

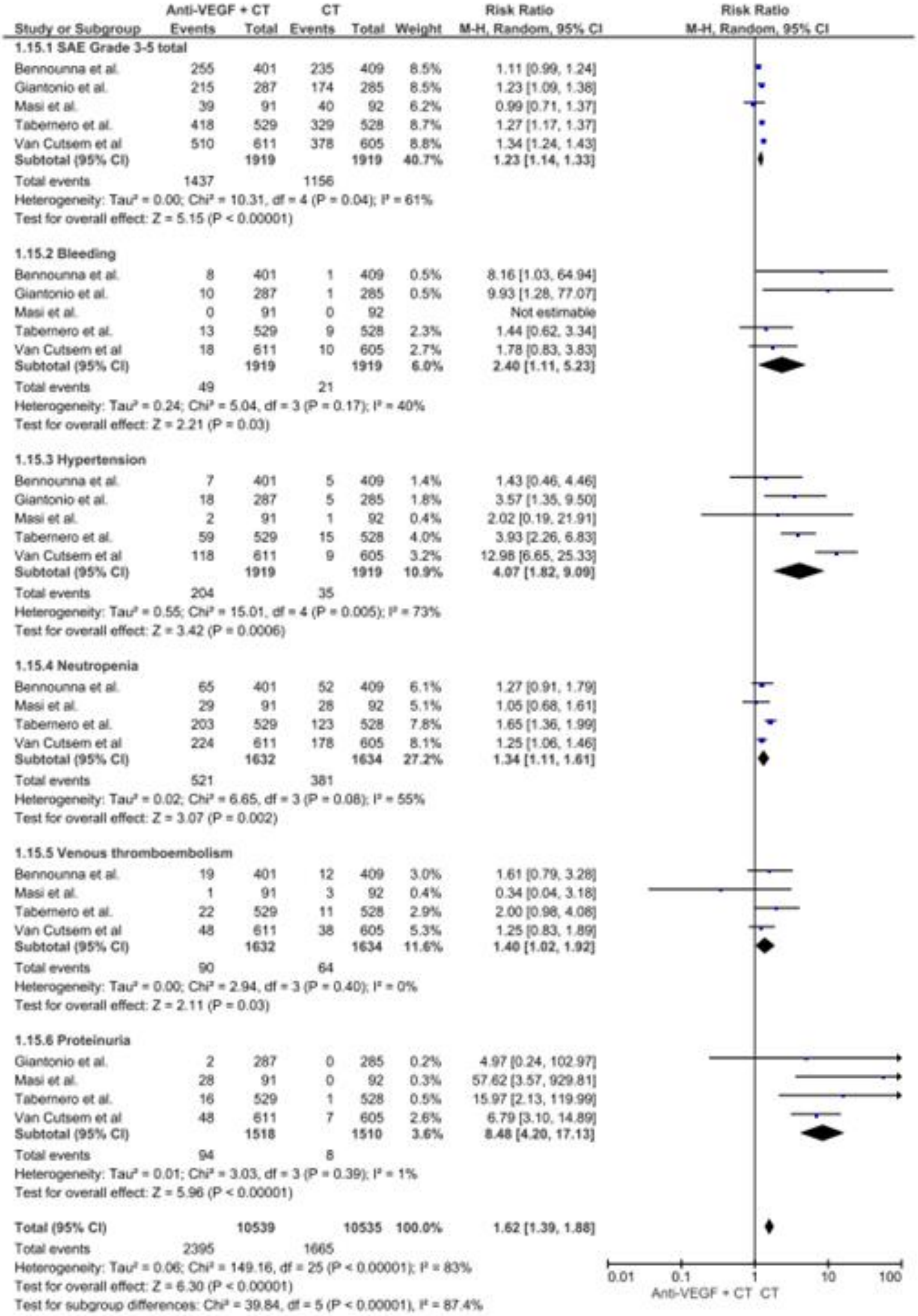

FigureS5

Study or Subgroup	Anti-VEGF + CT Events	Anti-VEGF + CT Total	CT Events	CT Total	Weight	Risk Ratio M-H, Fixed, 95% CI
**3.1.1 Diarrhea**						
Bennounna et al.	40	401	34	409	4.8%	1.20 [0.78, 1.86]
Masi et al.	6	91	6	92	0.9%	1.01 [0.34, 3.02]
Tabernero et al.	57	529	51	528	7.3%	1.12 [0.78, 1.60]
Van Cutsem et al	118	611	47	605	6.8%	2.49 [1.81, 3.42]
**Subtotal (95% CI)**		**1632**		**1634**	**19.7%**	**1.60 [1.31, 1.96]**
Total events	221		138			
Heterogeneity: Chi² = 13.59, df = 3 (P = 0.004); I² = 78%						
Test for overall effect: Z = 4.57 (P < 0.00001)						
**3.1.2 Vomiting**						
Bennounna et al.	14	401	13	409	1.8%	1.10 [0.52, 2.31]
Giantonio et al.	29	287	9	285	1.3%	3.20 [1.54, 6.64]
Masi et al.	2	91	2	92	0.3%	1.01 [0.15, 7.02]
Tabernero et al.	15	529	13	528	1.9%	1.15 [0.55, 2.40]
Van Cutsem et al	17	611	21	605	3.0%	0.80 [0.43, 1.50]
**Subtotal (95% CI)**		**1919**		**1919**	**8.3%**	**1.33 [0.95, 1.85]**
Total events	77		58			
Heterogeneity: Chi² = 8.52, df = 4 (P = 0.07); I² = 53%						
Test for overall effect: Z = 1.65 (P = 0.10)						
**3.1.3 Asthenia**						
Bennounna et al.	23	401	17	409	2.4%	1.38 [0.75, 2.54]
Tabernero et al.	61	529	41	528	5.9%	1.48 [1.02, 2.16]
Van Cutsem et al	103	601	64	605	9.1%	1.62 [1.21, 2.17]
**Subtotal (95% CI)**		**1531**		**1542**	**17.4%**	**1.54 [1.24, 1.91]**
Total events	187		122			
Heterogeneity: Chi² = 0.28, df = 2 (P = 0.87); I² = 0%						
Test for overall effect: Z = 3.94 (P < 0.0001)						
**3.1.4 Neutropenia**						
Bennounna et al.	65	401	52	409	7.4%	1.27 [0.91, 1.79]
Masi et al.	24	91	28	92	4.0%	0.87 [0.55, 1.38]
Tabernero et al.	203	529	123	528	17.6%	1.65 [1.36, 1.99]
Van Cutsem et al	224	611	178	605	25.6%	1.25 [1.06, 1.46]
**Subtotal (95% CI)**		**1632**		**1634**	**54.6%**	**1.35 [1.21, 1.51]**
Total events	516		381			
Heterogeneity: Chi² = 8.87, df = 3 (P = 0.03); I² = 66%						
Test for overall effect: Z = 5.28 (P < 0.00001)						
**Total (95% CI)**		**6714**		**6729**	**100.0%**	**1.43 [1.31, 1.56]**
Total events	1001		699			
Heterogeneity: Chi² = 33.97, df = 15 (P = 0.003); I² = 56%						
Test for overall effect: Z = 8.12 (P < 0.00001)						
Test for subgroup differences: Chi² = 2.82, df = 3 (P = 0.42), I² = 0%						

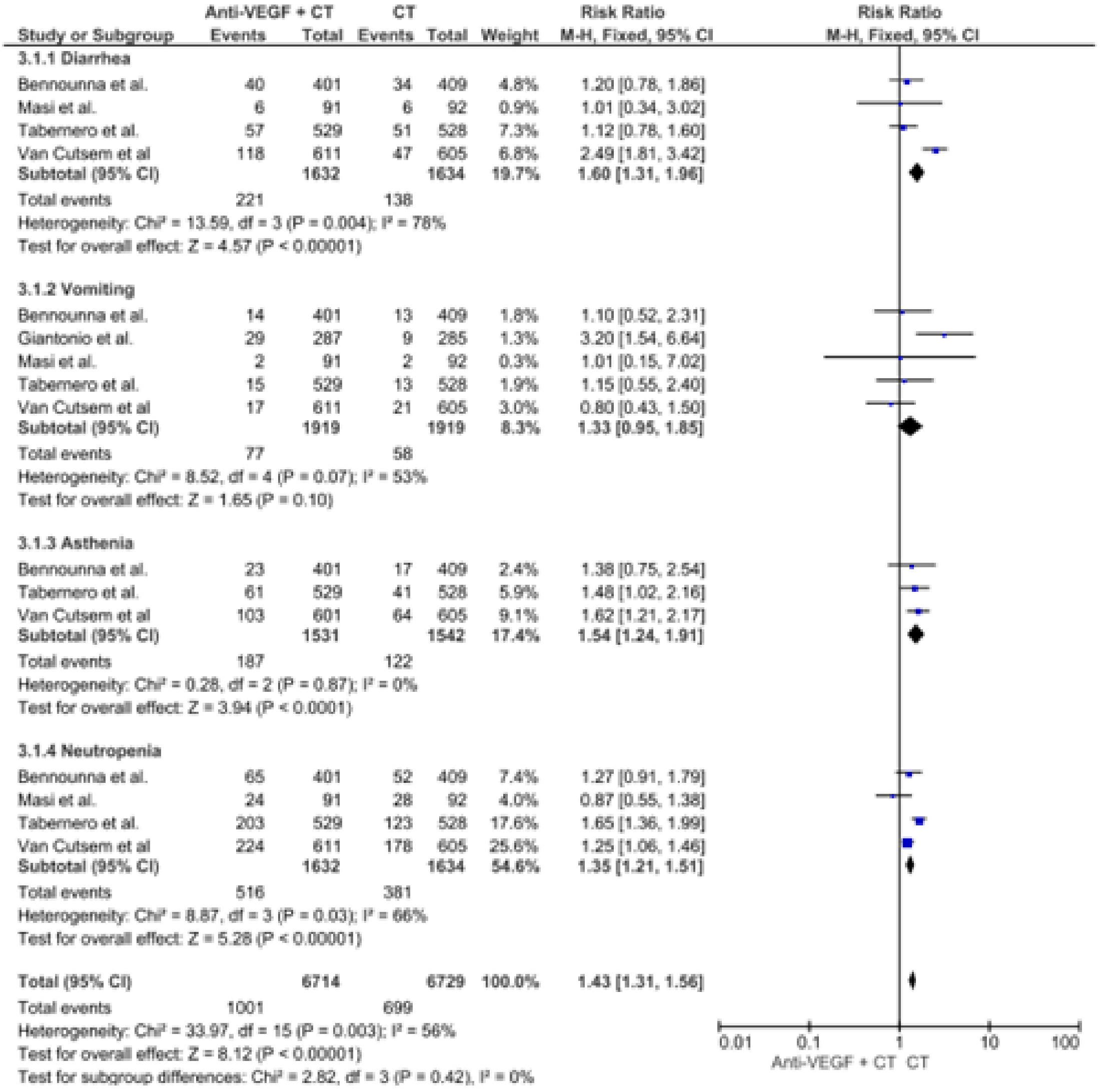

FigureS6

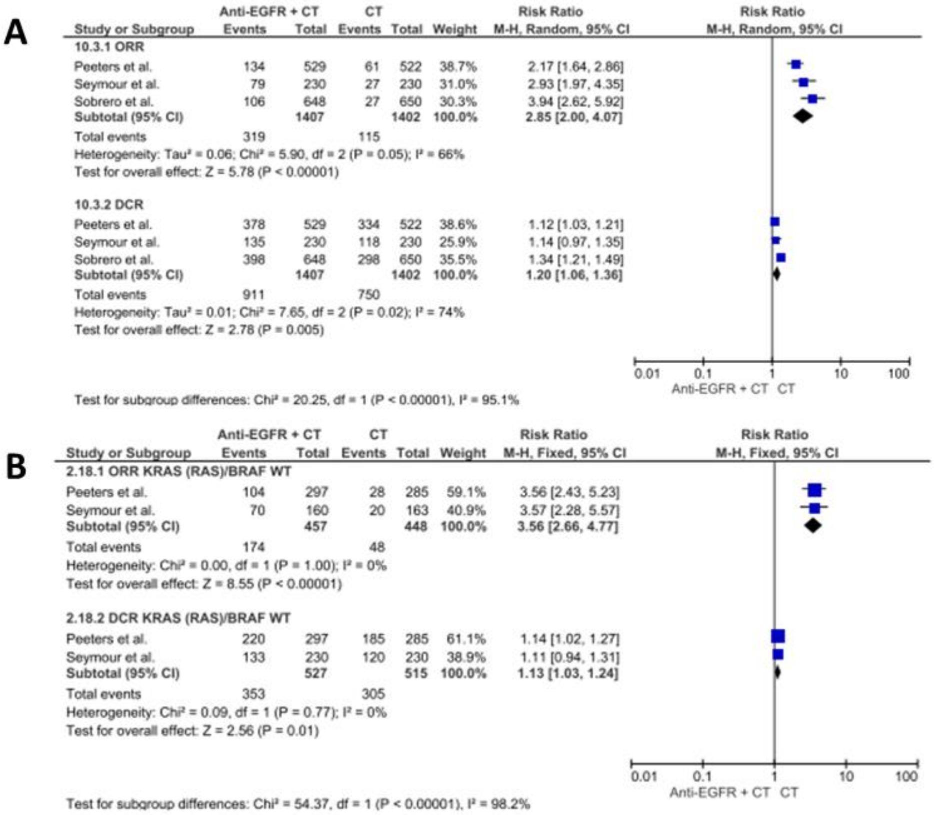
A
Anti-EGFR + CT
CT
Risk Ratio
Risk Ratio
Study or Subgroup Events Total Events Total Weight M-H, Random, 95% CI M-H, Random, 95% CI
10.3.1 ORR
Peeters et al. 134 529 61 522 38.7% 2.17 [1.64, 2.86]
Seymour et al. 79 230 27 230 31.0% 2.93 [1.97, 4.35]
Sobrero et al. 106 648 27 650 30.3% 3.94 [2.62, 5.92]
Subtotal (95% CI) 1407 1402 100.0% 2.85 [2.00, 4.07]
Total events 319 115
Heterogeneity: Tau² = 0.06; Chi² = 5.90, df = 2 (P = 0.05); I² = 66%
Test for overall effect: Z = 5.78 (P < 0.00001)
10.3.2 DCR
Peeters et al. 378 529 334 522 38.6% 1.12 [1.03, 1.21]
Seymour et al. 135 230 118 230 25.9% 1.14 [0.97, 1.35]
Sobrero et al. 398 648 298 650 35.5% 1.34 [1.21, 1.49]
Subtotal (95% CI) 1407 1402 100.0% 1.20 [1.06, 1.36]
Total events 911 750
Heterogeneity: Tau² = 0.01; Chi² = 7.65, df = 2 (P = 0.02); I² = 74%
Test for overall effect: Z = 2.78 (P = 0.005)
0.01 0.1 1 10 100
Anti-EGFR + CT CT
Test for subgroup differences: Chi² = 20.25, df = 1 (P < 0.00001), I² = 95.1%
B
Anti-EGFR + CT
CT
Risk Ratio
Risk Ratio
Study or Subgroup Events Total Events Total Weight M-H, Fixed, 95% CI M-H, Fixed, 95% CI
2.18.1 ORR KRAS (RAS)/BRAF WT
Peeters et al. 104 297 28 285 59.1% 3.56 [2.43, 5.23]
Seymour et al. 70 160 20 163 40.9% 3.57 [2.28, 5.57]
Subtotal (95% CI) 457 448 100.0% 3.56 [2.66, 4.77]
Total events 174 48
Heterogeneity: Chi² = 0.00, df = 1 (P = 1.00); I² = 0%
Test for overall effect: Z = 8.55 (P < 0.00001)
2.18.2 DCR KRAS (RAS)/BRAF WT
Peeters et al. 220 297 185 285 61.1% 1.14 [1.02, 1.27]
Seymour et al. 133 230 120 230 38.9% 1.11 [0.94, 1.31]
Subtotal (95% CI) 527 515 100.0% 1.13 [1.03, 1.24]
Total events 353 305
Heterogeneity: Chi² = 0.09, df = 1 (P = 0.77); I² = 0%
Test for overall effect: Z = 2.56 (P = 0.01)
0.01 0.1 1 10 100
Anti-EGFR + CT CT
Test for subgroup differences: Chi² = 54.37, df = 1 (P < 0.00001), I² = 98.2%

FigureS7

A

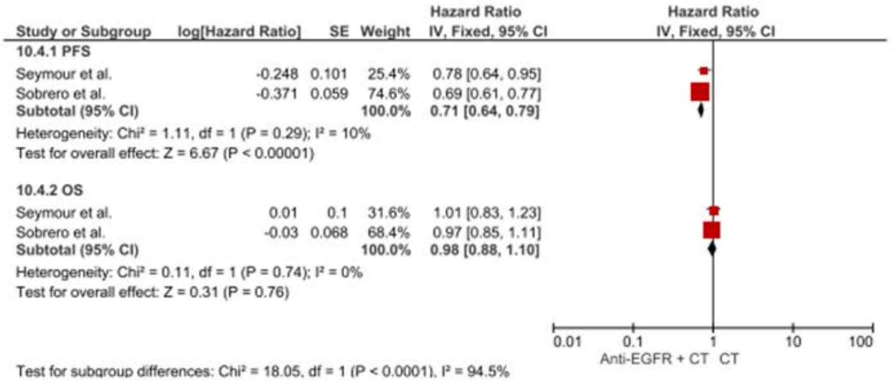
Study or Subgroup
log[Hazard Ratio]
SE
Weight
Hazard Ratio IV, Fixed, 95% CI
Hazard Ratio IV, Fixed, 95% CI
10.4.1 PFS
Seymour et al. -0.248 0.101 25.4% 0.78 [0.64, 0.95]
Sobrero et al. -0.371 0.059 74.6% 0.69 [0.61, 0.77]
Subtotal (95% CI) 100.0% 0.71 [0.64, 0.79]
Heterogeneity: Chi² = 1.11, df = 1 (P = 0.29); I² = 10%
Test for overall effect: Z = 6.67 (P < 0.00001)
10.4.2 OS
Seymour et al. 0.01 0.1 31.6% 1.01 [0.83, 1.23]
Sobrero et al. -0.03 0.068 68.4% 0.97 [0.85, 1.11]
Subtotal (95% CI) 100.0% 0.98 [0.88, 1.10]
Heterogeneity: Chi² = 0.11, df = 1 (P = 0.74); I² = 0%
Test for overall effect: Z = 0.31 (P = 0.76)
0.01
0.1
1
10
100
Anti-EGFR + CT CT
Test for subgroup differences: Chi² = 18.05, df = 1 (P < 0.0001), I² = 94.5%

B

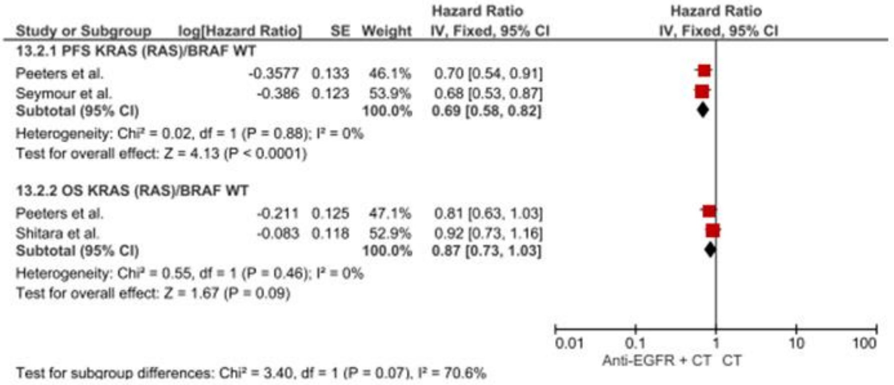
Study or Subgroup
log[Hazard Ratio]
SE
Weight
Hazard Ratio IV, Fixed, 95% CI
Hazard Ratio IV, Fixed, 95% CI
13.2.1 PFS KRAS (RAS)/BRAF WT
Peeters et al. -0.3577 0.133 46.1% 0.70 [0.54, 0.91]
Seymour et al. -0.386 0.123 53.9% 0.68 [0.53, 0.87]
Subtotal (95% CI) 100.0% 0.69 [0.58, 0.82]
Heterogeneity: Chi² = 0.02, df = 1 (P = 0.88); I² = 0%
Test for overall effect: Z = 4.13 (P < 0.0001)
13.2.2 OS KRAS (RAS)/BRAF WT
Peeters et al. -0.211 0.125 47.1% 0.81 [0.63, 1.03]
Shitara et al. -0.083 0.118 52.9% 0.92 [0.73, 1.16]
Subtotal (95% CI) 100.0% 0.87 [0.73, 1.03]
Heterogeneity: Chi² = 0.55, df = 1 (P = 0.46); I² = 0%
Test for overall effect: Z = 1.67 (P = 0.09)
0.01
0.1
1
10
100
Anti-EGFR + CT CT
Test for subgroup differences: Chi² = 3.40, df = 1 (P = 0.07), I² = 70.6%

FigureS8

Study or Subgroup	Anti-EGFR + CT Events	Anti-EGFR + CT Total	CT Events	CT Total	Weight	Risk Ratio M-H, Random, 95% CI
**2.15.1 SAE Grade 3-5 total**						
Peeters et al.	370	539	275	540	22.6%	1.35 [1.22, 1.49]
Seymour et al.	132	219	87	218	22.0%	1.51 [1.24, 1.84]
Sobrero et al.	396	638	274	629	22.6%	1.42 [1.28, 1.59]
**Subtotal (95% CI)**		**1396**		**1387**	**67.3%**	**1.40 [1.31, 1.50]**
Total events	898		636			
Heterogeneity: Tau² = 0.00; Chi² = 1.24, df = 2 (P = 0.54); I² = 0%						
Test for overall effect: Z = 9.56 (P < 0.00001)						
**2.15.2 Skin Toxicities**						
Peeters et al.	186	539	9	540	16.1%	20.71 [10.72, 39.99]
Seymour et al.	41	219	0	218	2.7%	82.62 [5.11, 1334.69]
Sobrero et al.	52	638	1	629	4.8%	51.27 [7.11, 369.70]
**Subtotal (95% CI)**		**1396**		**1387**	**23.6%**	**24.12 [13.11, 44.36]**
Total events	279		10			
Heterogeneity: Tau² = 0.00; Chi² = 1.62, df = 2 (P = 0.45); I² = 0%						
Test for overall effect: Z = 10.24 (P < 0.00001)						
**2.15.3 Hypomagnesemia**						
Peeters et al.	20	539	1	540	4.7%	20.04 [2.70, 148.77]
Sobrero et al.	9	638	1	629	4.5%	8.87 [1.13, 69.83]
**Subtotal (95% CI)**		**1177**		**1169**	**9.1%**	**13.49 [3.20, 56.81]**
Total events	29		2			
Heterogeneity: Tau² = 0.00; Chi² = 0.32, df = 1 (P = 0.57); I² = 0%						
Test for overall effect: Z = 3.55 (P = 0.0004)						
**Total (95% CI)**		**3969**		**3943**	**100.0%**	**3.56 [2.19, 5.79]**
Total events	1206		648			
Heterogeneity: Tau² = 0.27; Chi² = 142.27, df = 7 (P < 0.00001); I² = 95%						
Test for overall effect: Z = 5.13 (P < 0.00001)						
Test for subgroup differences: Chi² = 92.08, df = 2 (P < 0.00001), I² = 97.8%						

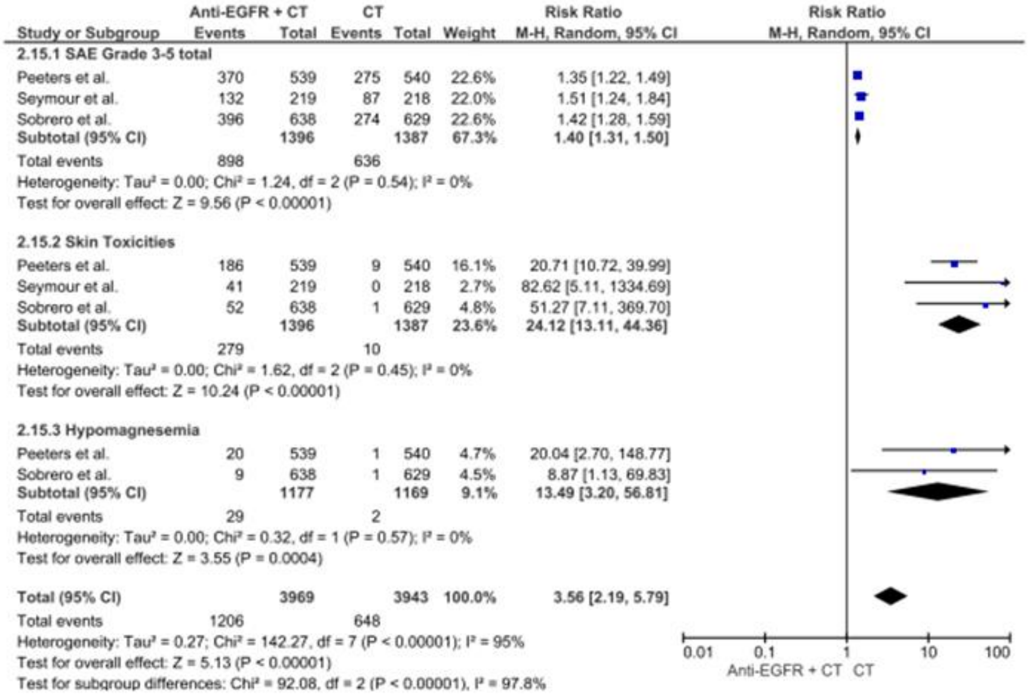

FigureS9

Study or Subgroup	Anti-EGFR + CT Events	Anti-EGFR + CT Total	CT Events	CT Total	Weight	Risk Ratio M-H, Fixed, 95% CI
**4.1.1 Diarrhea**						
Peeters et al.	73	539	40	540	4.6%	1.83 [1.27, 2.64]
Seymour et al.	64	219	39	218	4.5%	1.63 [1.15, 2.32]
Sobrero et al.	181	638	99	629	11.5%	1.80 [1.45, 2.24]
**Subtotal (95% CI)**		**1396**		**1387**	**20.7%**	**1.77 [1.50, 2.09]**
Total events	318		178			
Heterogeneity: Chi² = 0.26, df = 2 (P = 0.88); I² = 0%						
Test for overall effect: Z = 6.75 (P < 0.00001)						
**4.1.2 Vomiting**						
Peeters et al.	141	505	146	505	16.9%	0.97 [0.79, 1.17]
Seymour et al.	15	219	15	218	1.7%	1.00 [0.50, 1.99]
Sobrero et al.	33	638	34	629	4.0%	0.96 [0.60, 1.53]
**Subtotal (95% CI)**		**1362**		**1352**	**22.6%**	**0.97 [0.81, 1.15]**
Total events	189		195			
Heterogeneity: Chi² = 0.01, df = 2 (P = 1.00); I² = 0%						
Test for overall effect: Z = 0.38 (P = 0.70)						
**4.1.3 Asthenia**						
Peeters et al.	203	505	173	505	20.0%	1.17 [1.00, 1.38]
Sobrero et al.	29	638	28	629	3.3%	1.02 [0.61, 1.70]
**Subtotal (95% CI)**		**1143**		**1134**	**23.3%**	**1.15 [0.99, 1.35]**
Total events	232		201			
Heterogeneity: Chi² = 0.27, df = 1 (P = 0.61); I² = 0%						
Test for overall effect: Z = 1.79 (P = 0.07)						
**4.1.4 Neutropenia**						
Peeters et al.	91	539	111	540	12.8%	0.82 [0.64, 1.05]
Seymour et al.	48	219	27	218	3.1%	1.77 [1.15, 2.73]
Sobrero et al.	196	638	151	629	17.6%	1.28 [1.07, 1.53]
**Subtotal (95% CI)**		**1396**		**1387**	**33.5%**	**1.15 [1.00, 1.32]**
Total events	335		289			
Heterogeneity: Chi² = 12.10, df = 2 (P = 0.002); I² = 83%						
Test for overall effect: Z = 1.98 (P = 0.05)						
**Total (95% CI)**		**5297**		**5260**	**100.0%**	**1.24 [1.14, 1.34]**
Total events	1074		863			
Heterogeneity: Chi² = 39.85, df = 10 (P < 0.0001); I² = 75%						
Test for overall effect: Z = 5.34 (P < 0.00001)						
Test for subgroup differences: Chi² = 27.35, df = 3 (P < 0.00001), I² = 89.0%						

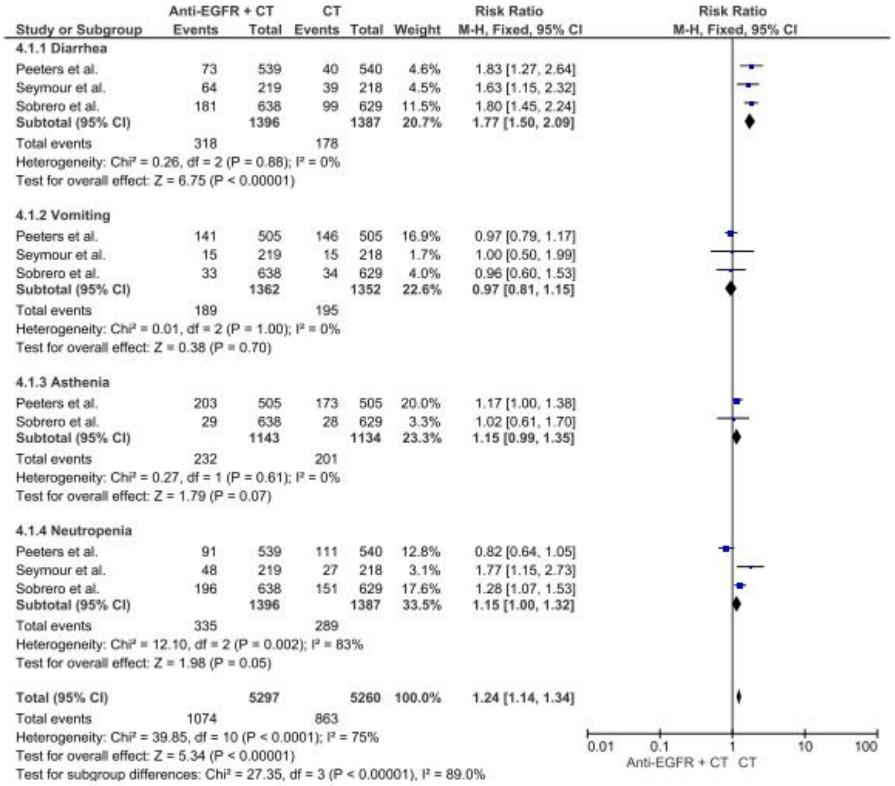

FigureS10

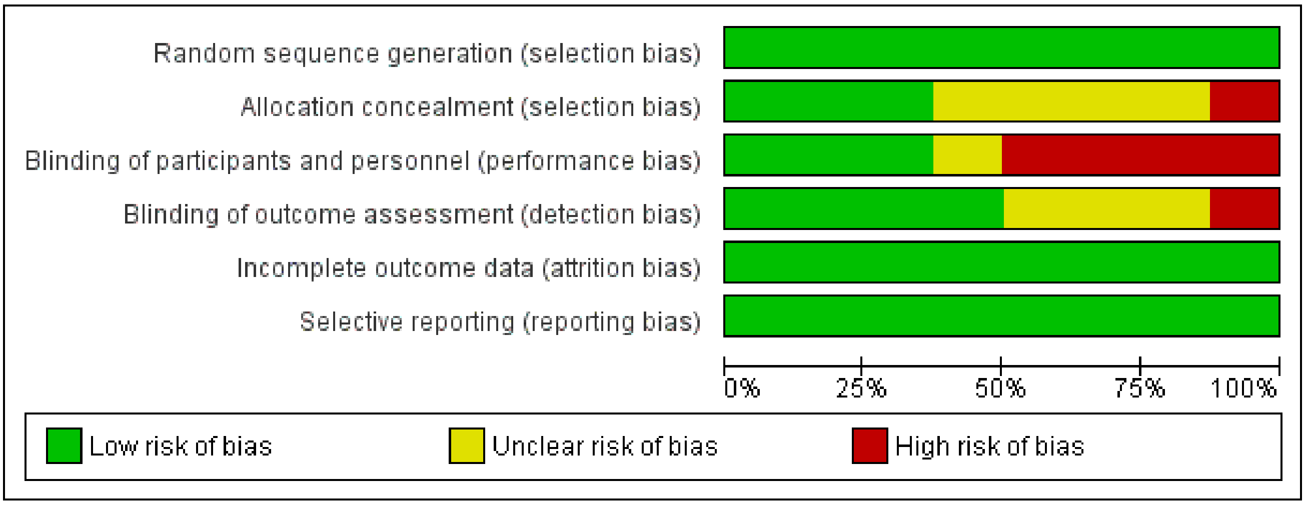

Random sequence generation (selection bias)
Allocation concealment (selection bias)
Blinding of participants and personnel (performance bias)
Blinding of outcome assessment (detection bias)
Incomplete outcome data (attrition bias)
Selective reporting (reporting bias)
0%
25%
50%
75%
100%
Low risk of bias
Unclear risk of bias
High risk of bias

FigureS11

Search Strategy

<u>ELECTRONIC SEARCH</u>

1. We used a search strategy Medline through Pubmed. The same search was then modified for searches in other databases (EMBASE, Controlled Cochrane Trial Register - CCTR).

The query was adapted from **Robinson &coll: [Robinson KA, Dickersin K. Development of a highly sensitive search strategy for the retrieval of reports of controlled trials using PubMed. International journal of epidemiology 2002;31:150-153].**

2. (("Colorectal Neoplasms"[Mesh] OR "colorectal cancer"[tiab] OR "CRC"[tiab]) AND ("second line"[tiab] OR "beyond first"[tiab] OR "progressing"[tiab]) AND ("bevacizumab"[tiab] OR "cetuximab"[tiab] OR "panitumumab"[tiab] OR "ramucirumab"[tiab] OR "aflibercept"[tiab]))

3. We used the free text strategy

Second line– advancedcolorectal cancer – Bevacizumab – Cetuximab – Panitumumab – Aflibercept - Ramucirumab

for trials on-line searches in Clinical Trials registers (www.clinicaltrials.gov)

3. We searched meta-analyses for any other published data concerningsecond line or advanced colorectal cancer OR The strategy in Pubmed for meta-analysis searching was:

"second line"[tiab] AND meta-analysis[pt]

"advanced colorectal cancer"[tiab] AND meta-analysis[pt]

("Colorectal Neoplasms"[Mesh] OR "CRC"[tiab] OR "colorectal"[tiab]) AND meta-analysis[pt]

FigureS12

Search Strategy

ELECTRONIC SEARCH

1. We used a search strategy Medline through Pubmed. The same search was then modified for searches in other databases (EMBASE, Controlled Cochrane Trial Register - CCTR).

The query was adapted from **Robinson &coll: [Robinson KA, Dickersin K. Development of a highly sensitive search strategy for the retrieval of reports of controlled trials using PubMed. International journal of epidemiology 2002;31:150-153].**

2. (("Colorectal Neoplasms"[Mesh] OR "colorectal cancer"[tiab] OR "CRC"[tiab]) AND ("second line"[tiab] OR "beyond first"[tiab] OR "progressing"[tiab]) AND ("bevacizumab"[tiab] OR "cetuximab"[tiab] OR "panitumumab"[tiab] OR "ramucirumab"[tiab] OR "aflibercept"[tiab]))

3. We used the free text strategy

Second line– advancedcolorectal cancer – Bevacizumab – Cetuximab – Panitumumab – Aflibercept - Ramucirumab

for trials on-line searches in Clinical Trials registers (www.clinicaltrials.gov)

3. We searched meta-analyses for any other published data concerningsecond line or advanced colorectal cancer OR The strategy in Pubmed for meta-analysis searching was:

"second line"[tiab] AND meta-analysis[pt]

"advanced colorectal cancer"[tiab] AND meta-analysis[pt]

("Colorectal Neoplasms"[Mesh] OR "CRC"[tiab] OR "colorectal"[tiab]) AND meta-analysis[pt]
